# Evaluation of an offline, artificial intelligence system for referable glaucoma screening using a smartphone-based fundus camera: a prospective study

**DOI:** 10.1038/s41433-023-02826-z

**Published:** 2023-12-13

**Authors:** Divya Parthasarathy Rao, Sujani Shroff, Florian M. Savoy, Shruthi S, Chao-Kai Hsu, Kalpa Negiloni, Zia Sultan Pradhan, Jayasree P V, Anand Sivaraman, Harsha L. Rao

**Affiliations:** 1 Remidio Innovative Solutions Inc, Glen Allen, VA USA; 2 Narayana Nethralaya Eye Hospital, Glaucoma Services, Bangalore, India; 3 Medios Technologies Pte Ltd, Singapore, Singapore; 4 Remidio Innovative Solutions Pvt Ltd, Bengaluru, India

**Keywords:** Optic nerve diseases, Vision disorders

## Abstract

**Background/Objectives:**

An affordable and scalable screening model is critical for undetected glaucoma. The study evaluated the performance of an offline, smartphone-based AI system for the detection of referable glaucoma against two benchmarks: specialist diagnosis following full glaucoma workup and consensus image grading.

**Subjects/Methods:**

This prospective study (tertiary glaucoma centre, India) included 243 subjects with varying severity of glaucoma and control group without glaucoma. Disc-centred images were captured using a validated smartphone-based fundus camera analysed by the AI system and graded by specialists. Diagnostic ability of the AI in detecting referable Glaucoma (Confirmed glaucoma) and no referable Glaucoma (Suspects and No glaucoma) when compared to a final diagnosis (comprehensive glaucoma workup) and majority grading (image grading) by Glaucoma specialists (pre-defined criteria) were evaluated.

**Results:**

The AI system demonstrated a sensitivity and specificity of 93.7% (95% CI: 87.6–96.9%) and 85.6% (95% CI:78.6–90.6%), respectively, in the detection of referable glaucoma when compared against final diagnosis following full glaucoma workup. True negative rate in definite non-glaucoma cases was 94.7% (95% CI: 87.2–97.9%). Amongst the false negatives were 4 early and 3 moderate glaucoma. When the same set of images provided to the AI was also provided to the specialists for image grading, specialists detected 60% (67/111) of true glaucoma cases versus a detection rate of 94% (104/111) by the AI.

**Conclusion:**

The AI tool showed robust performance when compared against a stringent benchmark. It had modest over-referral of normal subjects despite being challenged with fundus images alone. The next step involves a population-level assessment.

## Introduction

Glaucoma is a leading cause of global irreversible blindness. The prevalence is projected to increase from 76 million in 2020 to 111.8 million in 2040 [1]. Undetected glaucoma raises the risk of blindness and as the disease advances to late stages, the treatment and care cost significantly increase, posing a financial burden. This necessitates timely diagnosis and treatment [2, 3].

Glaucoma is a progressive degeneration of the optic nerve, with loss of retinal ganglion cells, thinning of the retinal nerve fibre layer, and progressive excavation of the optic disc [4]. Manual assessment of the optic nerve head (ONH), a crucial component of glaucoma diagnosis is labour-intensive and dependent on trained specialists. Fundus photography along with technology like Artificial Intelligence (AI) can help overcome this challenge.

AI helps triaging patients and ensuring emergent cases are referred appropriately to ophthalmologists [5, 6]. Global research for the development of an automated tool for glaucoma screening using fundus images has been promising [7, 8]. However, to the best of our knowledge, this is the first study validating an offline AI system in a prospective clinical study. Additionally, algorithms have typically been developed for bulky, expensive desktop fundus camera systems. This poses several challenges to widespread adoption. Requirements for stable internet connectivity for reporting and continuous power supply are barriers to accessibility in remote areas. To overcome these challenges, a novel AI for referable Glaucoma has been integrated offline on a validated smartphone-based, portable fundus camera. It can run in seconds without the need for internet or cloud-based inferencing [9]. The purpose of this study is to evaluate the performance of this novel system in detecting referable glaucoma on monoscopic fundus images.

## Materials and methods

A prospective, cross-sectional study was conducted at Narayana Nethralaya, a tertiary eye care centre, in South India between July 2021 and February 2022. The study adhered to the tenets of the Declaration of Helsinki and was approved by the Institute’s Ethics Committee (EC Ref No: C/2021/02/02). The study included consecutive patients visiting the clinic and written informed consent was obtained from all participants. The performance of the novel AI system (Medios AI-Glaucoma, Medios Technologies, Remidio Innovative Solutions, Singapore) was evaluated. The AI is integrated on a portable, smartphone-based fundus camera (Remidio NM-FOP 10, Remidio Innovative Solutions Pvt Ltd, Bengaluru, India). The AI system was compared against two benchmarks: standard of care i.e., final diagnosis provided by Glaucoma specialists following a thorough glaucoma evaluation as well as against the majority image grading diagnosis by three glaucoma specialists.

The study included consecutive, consenting patients above 18 years of age attending the glaucoma clinic with varying degrees of glaucomatous optic disc damage. In the control group, patients without glaucoma were recruited from the general ophthalmology clinics. Normal subjects were those who either walked into the general clinic for a routine evaluation or those who were referred from other hospitals or other departments of the same hospital for a glaucoma workup. The details of the exclusion criteria are presented in Supplementary Methods Section 1.

### Clinical evaluation

After recording the history and demographics, all participants underwent a complete ophthalmic evaluation including best corrected visual acuity (BCVA), slit lamp examination, intraocular pressure (IOP) by Goldmann Applanation Tonometer and gonioscopy using a 4-mirror goniolens. A dilated fundus evaluation included vertical cup-to-disc ratio (vCDR) measurement in increments of 0.05, and identification of other typical features of glaucomatous optic disc viz. neuroretinal rim thinning, notching, splinter haemorrhages, retinal nerve fibre layer defects and beta zone peripapillary atrophy. Following this, all patients underwent the imaging protocol described below by Optometrists with 1 year of experience.

#### Imaging protocol

A single 42-degree disc-centred image per eye was captured on the fundus on phone non-mydriatic (FOP NM-10) device (Remidio Innovative Solutions Pvt. Ltd, Bangalore, India). All acquired images were subjected to evaluation by the inbuilt image quality algorithm. The image quality assessment is based on the visualization of the optic disc, surrounding nerve fibre layer and 3rd-order vessels. If the image was of insufficient quality, the operator was alerted to take another image. The operator made a maximum of 2 attempts to get an image of sufficient quality.

Patients also underwent a single 30-degree disc-centred stereoscopic image captured on a standard tabletop fundus camera (Kowa NM WX-3D stereoscopic camera, Kowa, Japan). Following this, they underwent imaging of the optic disc using an SD-OCT device (Zeiss Cirrus SD-OCT, Dublin, CA). The optic nerve head and retinal nerve fibre layer were imaged using the optic disc cube scan.

Visual field examination (Humphrey visual field 24-2 or 10-2 programme) was performed in all new cases to establish the diagnosis of glaucoma and in confirmed cases if it was beyond 1 year since the last reliable fields.

All images were stored as JPEG files after removing patient identifiers and assigning a randomly generated unique numerical identifier linked to the participant’s study ID number.

### Final diagnosis

The glaucoma specialists (SS, SS, JVP) corroborated all the test results for a final diagnosis and categorized each eye into normal, glaucoma suspects, or glaucoma based on a predefined criteria [10] (Supplementary Methods Section 2). The worse eye diagnosis constituted the patient-level diagnosis. This was used as a reference standard against the binary output of the AI for referable glaucoma.

‘Referable glaucoma’ referred to those with glaucoma and ‘No referable glaucoma’ included glaucoma suspects and normal.

### Fundus image quality control and grading

All the images captured using the Kowa stereoscopic camera and the FOP-NM 10 device were evaluated by three fellowship-trained glaucoma specialists (SS, SS, JVP). They were masked to the clinical examination details, investigational reports as well as each other’s grading. The graders initially evaluated the quality of the images as excellent, acceptable, or insufficient based on the criteria mentioned in Supplementary Methods Section 3. Excellent and acceptable grades qualified as sufficient image quality. A predefined criterion from previous population studies was used by the specialists for making a provisional diagnosis (unlikely glaucoma, disc suspects or likely glaucoma) of glaucoma as mentioned in Supplementary Methods Section 4 [11–14]. Glaucoma severity was determined based on visual field MD as per Hodapp-Parish and Anderson criteria. Mean Deviation (MD) less than –6 dB was early, −6 to –12 dB was moderate and worse than –12 dB was defined as severe disease [15].

‘Referable’ glaucoma referred to those with likely glaucoma and ‘No referable glaucoma’ included disc suspects and unlikely glaucoma.

### Automated referable glaucoma AI detection system

The AI system consists of two main components: a cup and disc segmentation model and a binary classification model. The segmentation model has been described and externally validated in a prospective study [16]. The classification model segregates images with glaucoma from suspects and normal eyes. It has been trained using 6674 images. 1813 (27.2%) were glaucoma, 1142 (17.1%) were suspects and 3719 (55.7%) were normal eyes. 4373 images (65.5%) were captured using the Remidio FOP (target deployment device), and 2301 (34.5%) using desktop fundus cameras. 5082 images (76.1%) were captured on a South Asian population, and 1592 (23.9%) on a Caucasian population. The model uses a ResNet-50 architecture and was pre-trained on the ImageNet dataset. Additionally, the datasets were carefully curated during development such that there was no overlap of patient data during training and testing. Two other assistive AI models were trained. The first is a quality check which outputs an indication of sufficient image quality for a reliable glaucoma diagnosis. The second is a disc localization model. It detects the location of the centre of the disc in the retinal image. The disc coordinates are used to crop a region of interest around the disc. This is a pre-processing step for the two main AI models (segmentation and classification algorithms). Supplementary flowchart summarizes the different elements of the AI system. This study was conducted following AI development and internal testing.

The images of all the participants were analysed using the AI tool. The AI graded the images as Referrable or No Referable Glaucoma. Referrable glaucoma included those with likely glaucoma requiring immediate referral and no referable glaucoma included disc suspects and no glaucoma. The AI also categorizes images with high VCDR (vCDR 0.7–0.85) and no other glaucomatous disc changes as ‘high VCDR (disc suspect)’ with a non-urgent referral to the ophthalmologist.

The primary outcome measure was the diagnostic ability of AI in detecting referable Glaucoma when compared to a final diagnosis made by a glaucoma specialist following a complete glaucoma evaluation. The secondary outcome measures were (1) diagnostic ability of the AI when compared against a majority image grading diagnosis provided by glaucoma specialists (2) comparing the image quality and diagnostic accuracy in the detection of referable glaucoma using monoscopic and stereoscopic fundus camera images and (3) repeatability analysis of the AI output.

### Sample size calculation

The minimum required sample calculated to detect the sensitivity of 80% (and addressing a specificity of 80%) with a precision of 10% was 154 patients. This incorporates a 40% prevalence of referable Glaucoma and a 95% confidence level. A sample size of 200 patients was also sufficient to measure rate of discordance in referrable glaucoma between the AI software and glaucoma specialist from the true rate of discordance by ≤8% assuming a true discordance rate ranging between 10 and 50%, and sensitivity of at least 80%. We aimed for at least 250 patients for the current study assuming a 25% attrition due to incomplete tests, dropouts and quality/reliability issues from various devices.

### Statistical analysis

A patient-level analysis included the diagnosis of the worse eye for the presence of referable glaucoma. A 2*2 confusion matrix was used to compute the sensitivity and specificity of the AI. Additional metrics included the likelihood ratios (LR) and accuracy along with Wilson’s 95% Confidence Intervals (CI). A weighted kappa statistic (pairwise) was used to determine the interobserver agreement. Kappa of 0–0.20 was considered as slight agreement, 0.21–0.40 as fair, 0.41–0.60 as moderate, 0.61–0.80 as substantial, and 0.81–1 as almost perfect agreement [17]. Image quality of the monoscopic and stereoscopic images was assessed on a majority grading basis as a proportion of sufficient (excellent and acceptable images) and insufficient quality images for a reliable glaucoma diagnosis. Additionally, the AI image quality algorithm was evaluated by image-ability, defined as the percentage of images determined as sufficient quality by the AI within the subset of images deemed sufficient by the graders [18]. All data was stored in Microsoft Excel and was analysed using Python 3.7, as well as the NumPy 1.21 and SciPy 1.7 libraries.

## Results

**Fig. 1:**
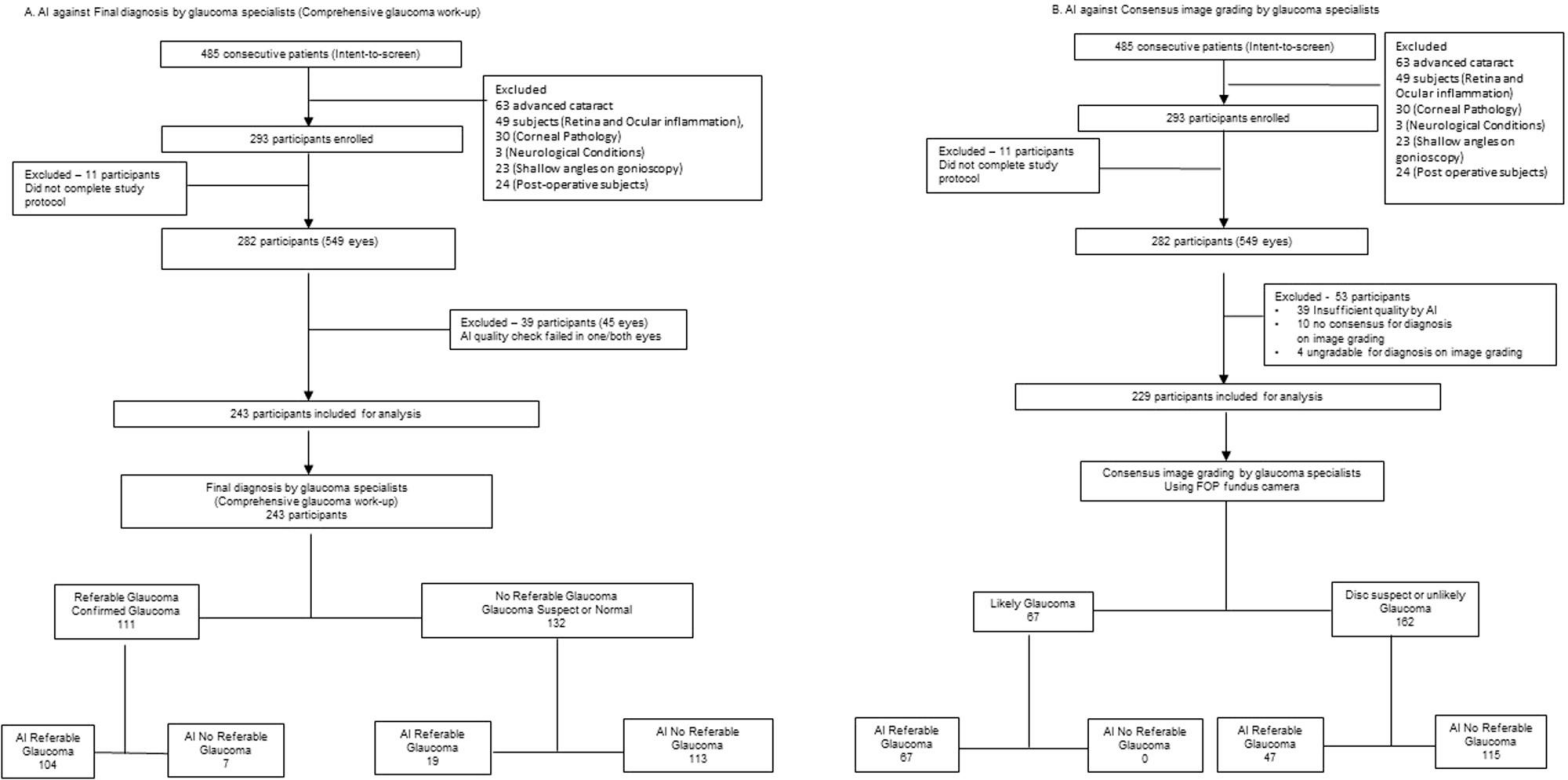
Flow diagram for participant disposition in medios automated referable glaucoma detection artificial intelligence system study.

A total of 485 consecutive patients were screened and 293 participants were recruited. The mean age was 59 ± 12 years (range, 21, 83), 92% were greater than 40 years and 49% (*n* = 144) were female. There were 242 eyes with early to moderate cataract and 143 pseudophakia included in the study. 11 subjects were excluded as they did not complete the study protocol. Of the 282 participants (549 eyes), 39 were excluded (45 eyes) due to failed AI image quality in one or both eyes (image capture technology failure). 243 participants were included in the final analysis (Fig. 1).

### Comparison of AI output against final diagnosis following a comprehensive glaucoma workup

**Fig. 2:**
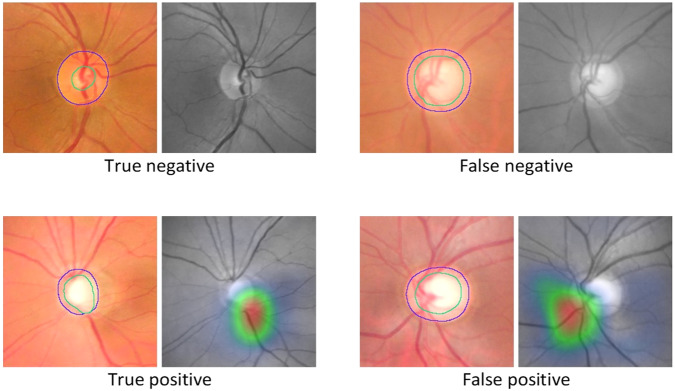
Representative outputs of the AI system along with Class Activation Maps (CAMs) for the positive cases.

Following a thorough glaucoma evaluation of 243 subjects, 111 subjects (45.67%), were diagnosed to have glaucoma, 56 (23.05%) were glaucoma suspects and 76 (31.28%) were normal. The AI system accurately detected glaucoma in 104 out of the 111 subjects. The sensitivity and specificity were 93.7% (95% CI: 87.6–96.9%) and 85.6% (95% CI: 78.6 – 90.6%), respectively in the detection of referable glaucoma. The true negative rate in definite non-glaucoma cases (i.e., the proportion of patients being normal on thorough glaucoma evaluation which have been correctly identified as no glaucoma by the AI) was 94.7% (95% CI: 87.2–97.9%). There were 7 (6.3%) false negative glaucoma cases (three diagnosed as disc suspect and four as normal by AI). On a closer evaluation, 4 were found to be early, 3 were found to be moderate glaucoma and none with advanced glaucoma. There were 19 (14.4%) false positive cases that included 15 diagnosed as disc suspects and 4 determined to be normal by the specialists. The performance of the AI system is summarized in Table 1. Representative outputs of correctly (True Negative and True Positive) and incorrectly (False Negative and False Positive) identified images by the algorithm along with class activations maps for the positive images are presented in Fig. 2.

**Table 1:** Referable Glaucoma AI performance when compared against final diagnosis following comprehensive glaucoma evaluation.

			Glaucoma specialist diagnosis (*n* = 243)		
			Confirmed Glaucoma	Glaucoma Suspects	Normal
(a) Confusion matrix—AI system versus final diagnosis by Glaucoma specialists					
AI Diagnosis	Referable Glaucoma		104 (43%)	15 (6%)	4 (2%)
	No Referable Glaucoma	Disc Suspect	3 (1%)	19 (8%)	18 (7%)
		No Glaucoma	4 (2%)	22 (9%)	54 (22%)
		Total	111	56	76
(b) Confusion matrix—AI system versus final diagnosis based on Glaucoma severity (HAP criteria [15]) by the specialists (*N* = 111 confirmed glaucoma)					
			Glaucoma severity diagnosis by specialists		
			Early	Moderate	Advanced
AI Diagnosis	Referable Glaucoma		26	22	56
	No Referable Glaucoma	Disc Suspect	2	1	
		No Glaucoma	2	2	
(c) AI performance in the detection of Referable Glaucoma (Final diagnosis)					
Sensitivity			93.7% (95% CI: 87.6–96.9%)		
Specificity			85.6% (95% CI: 78.6–90.6%)		
Accuracy			89.3% (95% CI: 84.7–92.9%)		
Positive likelihood ratio			6.51 (95% CI: 4.28–9.90)		
Negative likelihood ratio			0.07 (95% CI: 0.04–0.15)		
Recall- No glaucoma			94.7% (95% CI: 87.2–97.9%)

### Comparison of monoscopic images (FOP NM-10) vs stereoscopic images (Kowa) for image quality and agreement for glaucoma diagnosis

282 participants had a total of 549 images (15 one-eyed subjects), which were graded by three blinded, glaucoma specialists. Of these, 45 images failed AI quality check and 504 images (from 275 participants) were of sufficient quality. (Supplementary Table 1). 493/504 (97.8%) images on the FOP and 496/503 (98.6%) images on the Kowa were deemed to be of sufficient quality for a reliable glaucoma grading by the graders. Table 2 describes the details of image quality analysis between the two systems. The three specialists had consensus on 95.8 to 96.7% of the images on both systems for making a diagnosis. A pair-wise kappa analysis was between 0.72–0.74 on the FOP and 0.70–0.79 on the Kowa (Table 2).

**Table 2:** Comparison of monoscopic images (FOP NM-10) vs stereoscopic images (Kowa) for image quality and agreement for glaucoma diagnosis.

		Image grading by specialists	
		Monoscopic images (FOP NM-10) *N* = 504 images	Stereoscopic images (Kowa) *N* = 503 images
	Excellent	372 (73.8%)	413 (82.1%)
	Acceptable	121 (24.0%)	83 (16.5%)
Quality of fundus images	Total sufficient quality	493 (97.8%)	496 (98.6%)
	Insufficient	8 (1.6%)	3 (0.6%)
	No consensus	3 (0.6%)	4 (0.8%)
Consensus amongst graders on diagnosis (Patient level)	Yes	229 (95.8%)	233 (96.7%)
	No	10 (4.2%)	8 (3.3%)
Inter-grader agreement (Cohens kappa, Glaucoma diagnosis)	Ophthalmologist 1 and 2	0.72	0.70
	Ophthalmologist 1 and 3	0.74	0.76
	Ophthalmologist 2 and 3	0.73	0.79

Evaluation of the image quality AI on the FOP: 56 out of 549 FOP images received an insufficient image quality label by either the AI or the image graders or had no consensus. The graders identified 23 images as ungradable, and 4 had no consensus. Thus, 522 images were deemed to have sufficient quality by the graders. Amongst them, an additional 29 (5.6%) received an insufficient image quality from the AI. Thus, image-ability, was high at 94.4% (493/522). Supplementary Table 1 provides a summary of the results.

### Comparison of AI against image grading by Glaucoma specialists on FOP NM-10 Fundus camera

Of 282 subjects, 229 were included for analysis of AI performance against image grading on FOP (Fig. 1). The specialists detected 60% (67/111) of true glaucoma cases by grading just fundus images versus a detection rate of 94% (104/111) by the AI. Table 3 details the performance of the algorithm against image grading.

**Table 3:** Referable Glaucoma AI performance when compared against image grading using FOP and Kowa fundus camera images.

			Image grading using FOP fundus camera (*n* = 229)			Image grading using Kowa fundus Camera (*n* = 233)		
			Likely Glaucoma	Disc Suspect	Unlikely Glaucoma	Likely Glaucoma	Disc Suspect	Unlikely Glaucoma
AI Diagnosis	Referable Glaucoma		67 (29%)	26 (11%)	21 (9%)	77 (33%)	24 (10%)	17 (7%)
	No Referable Glaucoma	Disc Suspect	0	13 (6%)	25 (11%)	0	12 (5%)	25 (11%)
		No Glaucoma	0	6 (3%)	71 (31%)	0	5 (2%)	73 (31%)

(b) AI performance in the detection of Referable Glaucoma (consensus image grading)		
	Image grading using FOP fundus camera	Image grading using Kowa camera
Sensitivity	100 % (95% CI: 94.6–100%)	100% (95% CI: 95.2–100%)
Specificity	71.0% (95% CI: 63.6–77.4%)	73.7% (95% CI: 66.3–80.0%)
Accuracy	79.5% (95% CI: 73.7–84.5%)	82.4% (95% CI: 76.9–87.1%)
Positive likelihood ratio	3.45 (95% CI: 2.71–4.39)	3.80 (95% CI: 2.93–4.95)
Negative likelihood ratio	0.00	0.00
Recall- no glaucoma	82.1 (95% CI: 74.1–88.0%)	85.2% (95% CI: 77.6–90.6%)

### Repeatability

A repeatability analysis was performed on a subset of 32 eyes. This included 15 eyes with a final diagnosis of glaucoma and 17 eyes with a final diagnosis of no glaucoma randomly chosen. Each eye was imaged three times, with all three resulting images being fed to the AI independently. For 30/32 eyes, the output of the AI was identical amongst all three runs. The two cases with disagreements consisted of one glaucoma and one normal case. The repeatability was thus 93.75%.

## Discussion

An alarming trend shows more than 90% of glaucoma in the community being undetected in developing nations. Additionally, more than 50% have advanced disease and nearly 20% are blind at the time of diagnosis [19–21]. Compounding this problem is an acute shortage of glaucoma specialists. Studies in developing countries have shown that Glaucoma screening can be cost-effective [22, 23]. This necessitates a tool that leverages technologies like AI to address the inequities in screening making it effective and labour-sparing in at least the high-risk populations. Adding to the challenge is the absence of objective, standardized criteria that is universally agreed upon for diagnosing suspicious discs. This leads to subjectivity in not only the diagnosis but also the management of glaucoma suspects and early disease. We aimed to develop a novel, affordable screening tool using fundus images that can accurately identify those well-established glaucoma cases who are undetected in the community. They would benefit from immediate referral and management or would otherwise go blind. Due to the low prevalence of the disease, the algorithm was developed with the idea of maximizing the sensitivity for those with established glaucoma while maintaining a high specificity to avoid an over-referral or alarm amongst normal subjects.

Generally, structural changes in the optic nerve head (ONH) like neuroretinal rim abnormalities and enlargement of ONH excavation precede functional loss detectable on visual field assessment [4]. Hence, these morphological changes are considered early biomarkers for glaucomatous optic neuropathy (GON). Fundus cameras capturing monoscopic colour images, red-free images or stereo images of the optic disc and RNFL have been widely used to detect structural changes and monitor glaucoma [24]. Stereoscopic imaging has better visualization of ONH morphology due to depth perception. However, these systems are large, unwieldy and expensive. In the current study, while the proportion of excellent quality images on the traditional desktop stereo camera was higher (82.1% Kowa vs 73.8% on FOP), the overall sufficient quality images for a reliable glaucoma diagnosis between the monoscopic (97.8% sufficient quality) and stereoscopic fundus camera (98.6% sufficient quality) were similar. While the specialists identified a marginally higher number of likely glaucoma cases on the stereoscopic camera (33% on Kowa vs 29% on FOP), the AI performance on the smartphone camera was unaffected when compared against imaging grading on either device. The AI correctly detected all the glaucoma cases identified by the specialists on either device (Sensitivity of AI 100% against both for image-based grading). This shows that the monoscopic fundus camera integrated with the robust AI has the potential for Glaucoma screening. It has significant public health relevance as it is easier to capture images on a portable fundus camera that is a fraction of the cost of a high-end expensive stereo fundus camera. This highlights the potential application of the AI system in a population-based setting to be used either independently or along with teleophthalmology as a clinical assist tool.

To present the accuracy of the AI system in referable glaucoma detection, we compared the AI system against two benchmarks: final diagnosis following a thorough glaucoma evaluation (standard of care) and image grading by glaucoma specialists on the same set of patients. This provides a better understanding of the reliability of image grading for glaucoma diagnosis. The AI system had a sensitivity and specificity of 93.7% and 85.6%, respectively, in comparison against standard of care. The 7 false negative cases were early (4) and moderate (3) glaucoma cases with no advanced case being missed. False positives (19 cases, 14.4%) included both disc suspects and normal cases being flagged as glaucoma by the AI. While the specificity seems relatively low, it is essential to recognize that the false positives were primarily disc suspects (15/19 cases) who would require a glaucoma workup and periodic yearly monitoring while not requiring urgent attention. This could also be attributable to a larger proportion of suspicious discs being evaluated in a tertiary centre. Interestingly, only 4 out of 76 normal subjects were considered referable glaucoma. Hence, the true negative rate in the definite non-glaucoma cases, or in other words, accurately identifying those without glaucoma was 94.7% (72/76; 95% CI: 87.2–97.9%). This is critical in a disease like glaucoma where minimal over-referral of normal subjects is pivotal to preventing overburdening of an already stretched health care system. On a closer evaluation, three of these subjects had a higher-than-average vCDR. It must be noted that at the population level, the prevalence of disease is low and hence the distribution of those with no glaucoma will be significantly higher. Hence, population-level specificity is to be evaluated in a subsequent study. Direct comparison to other global research groups is challenging due to differences in disease definitions, comparison standards, models utilized and the population in which the algorithm was validated. However, our model performed on par with other groups despite having a more difficult benchmark of comparison. Supplementary Table 2 summarizes various glaucoma detection studies using AI and Deep Learning on fundus photographs [25–33]. In the future, to improve the accuracy of the deep learning algorithm and further reduce the false negatives, more data coming from early-moderate cases along with corresponding OCT information during development will be useful.

The AI had a sensitivity of 100% for referable Glaucoma when compared against the consensus image grading of three glaucoma specialists. Inspecting the specificity of 71% (47 false positives) against image grading, we observed that 55% (26 cases) of false positives were graded as disc suspects and 21 as unlikely glaucoma by the specialists. Interestingly, 18 among these 26 cases and 10 out of 21, respectively, were diagnosed as having glaucoma on full evaluation contributing to the apparently low specificity on image grading. Overall, the specialists detected 60% (67/111) of true glaucoma cases by grading just fundus images versus a detection rate of 94% (104/111) by the AI on the same images. We hypothesize that the algorithm may have learnt, during the development phase, to identify subtle structural changes on fundus images that may not be very evident to the human eye. It shows great promise as a screening tool. However, it is important to address that this AI system cannot replace an ophthalmologist in decision-making on the final diagnosis for glaucoma. The gold standard still remains an ophthalmologist’s diagnosis based on history, detailed clinical exam along with interpretation of multimodal testing (structural and functional assessment) while excluding other causes of optic neuropathy.

Most AI algorithms require fast internet connectivity and high computational power for reporting [25, 30]. Additionally, they are developed to work on high-end, costly tabletop fundus cameras limiting their utility in resource-constrained settings [18, 34]. The current AI system utilizes lightweight deep neural network architectures that are deployed on a low-cost, smartphone-based fundus camera without compromising on efficiency or accuracy, which is a key highlight. This makes the implementation of screening programmes in the outreach practical. To the best of our knowledge, it is the first offline, on-the-edge software for screening eye conditions such as Diabetic Retinopathy and Glaucoma that can give a report within a few seconds without the need for internet connectivity [35–37]. The portable design of this device with its embedded AI system makes it user-friendly and can be used by minimally trained health workers [38, 39].

Strengths of the study: This is the first prospective study evaluating an offline AI for screening referable glaucoma using smartphone-based monoscopic fundus images and showing promising performance. Additionally, the accuracy has been determined against two benchmarks: comprehensive evaluation and image grading by glaucoma specialists. The diagnostic criteria for both evaluations were standardized to lower the chance of subjective assessment. A stringent assessment against the gold standard despite the AI being presented with fundus images allows for a robust evaluation of the AI system. Adequate sample size with a good distribution of disease spectrum from no glaucoma to suspects to confirmed glaucoma ensured a thorough evaluation.

Limitations of the study: The performance of the AI has been evaluated in a South Asian population. To understand the generalizability of the model across geographies, a multi-ethnic validation will be essential. The purpose of this study was to evaluate the performance of this novel algorithm in a tertiary glaucoma centre (controlled setting) given the necessity to establish a robust ground truth with a comprehensive glaucoma work-up requiring several diagnostic modalities (clinical, structural and functional). Expectedly, the number of glaucoma and suspect cases was higher. While the results are promising, further evaluation in a real-world community setting is essential to understand whether the results can be extrapolated to a population setting with true disease prevalence, which is currently underway.

In conclusion, the novel AI integrated on a portable fundus camera can have a significant impact in screening for referable glaucoma. It can enable healthcare workers in low resource environments to screen and help break barriers to eyecare access. While this tool shows promising results, it is essential to start working towards strengthening the existing healthcare system to take on the additional burden of patients being moved into the referral care pathway. This will ensure that improved patient outcome is ultimately achieved.

## Summary

### What was known before

- Currently, available tools are not ideal for glaucoma screening.
- Global research has found promising utility in using AI algorithms on fundus images for screening. However, they have typically been developed for bulky, expensive desktop fundus cameras with cloud-based inferencing that pose several challenges for widespread adoption.
- There is also a lacuna in terms of a prospective clinical study to validate these solutions against a gold standard diagnosis of glaucoma.

### What this study adds

- A novel, offline AI deployed on a portable, affordable and validated smartphone-based fundus camera shows a robust performance in detecting referable glaucoma in a prospective clinical study.
- Comparison against gold standard diagnosis demonstrates the true potential of the solution to triage undetected glaucoma cases to the referral care pathway.
- It holds promise for a scalable solution as it provides instant reports and overcomes several barriers associated with current technology for screening in the community.

### Supplementary information

Supplementary methods

Supplementary Flowchart

Supplementary Table 1

Supplementary table 2

## Data Availability

The data can be shared upon reasonable request to the corresponding author

## References

[1] Tham YC, Li X, Wong TY, Quigley HA, Aung T, Cheng CY (2014). Global prevalence of glaucoma and projections of glaucoma burden through 2040: a systematic review and meta-analysis. Ophthalmology.

[2] Zhang Y, Jin G, Fan M, Lin Y, Wen X, Li Z (2019). Time trends and heterogeneity in the disease burden of glaucoma, 1990–2017: a global analysis. J Glob Health.

[3] Delgado MF, Abdelrahman AM, Terahi M, Miro Quesada Woll JJ, Gil-Carrasco F, Cook C (2019). Management of glaucoma in developing countries: challenges and opportunities for improvement. Clinicoecon Outcomes Res.

[4] Weinreb RN, Aung T, Medeiros FA (2014). The pathophysiology and treatment of glaucoma: a review. JAMA.

[5] Gunasekeran DV, Wong TY (2020). Artificial intelligence in ophthalmology in 2020: a technology on the cusp for translation and implementation. Asia Pac J Ophthalmol (Philos).

[6] Hogarty DT, Mackey DA, Hewitt AW (2019). Current state and future prospects of artificial intelligence in ophthalmology: a review. Clin Exp Ophthalmol.

[7] Mayro EL, Wang M, Elze T, Pasquale LR (2020). The impact of artificial intelligence in the diagnosis and management of glaucoma. Eye..

[8] Mursch-Edlmayr AS, Ng WS, Diniz-Filho A, Sousa DC, Arnold L, Schlenker MB (2020). Artificial intelligence algorithms to diagnose glaucoma and detect glaucoma progression: translation to clinical practice. Transl Vis Sci Technol.

[9] Varshney T, Parthasarathy DR, Gupta V (2021). Artificial intelligence integrated smartphone fundus camera for screening the glaucomatous optic disc. Indian J Ophthalmol.

[10] Mariottoni EB, Jammal AA, Berchuck SI, Shigueoka LS, Tavares IM, Medeiros FA (2021). An objective structural and functional reference standard in glaucoma. Sci Rep.

[11] Foster PJ, Buhrmann R, Quigley HA, Johnson GJ (2002). The definition and classification of glaucoma in prevalence surveys. Br J Ophthalmol.

[12] Iwase A, Suzuki Y, Araie M, Yamamoto T, Abe H, Shirato S (2004). The prevalence of primary open-angle glaucoma in Japanese: the Tajimi Study. Ophthalmology.

[13] He M, Foster PJ, Ge J, Huang W, Zheng Y, Friedman DS (2006). Prevalence and clinical characteristics of glaucoma in adult Chinese: a population-based study in Liwan District, Guangzhou. Invest Ophthalmol Vis Sci.

[14] Topouzis F, Wilson MR, Harris A, Anastasopoulos E, Yu F, Mavroudis L (2007). Prevalence of open-angle glaucoma in Greece: the Thessaloniki Eye Study. Am J Ophthalmol.

[15] Hodapp E, Parrish RK II, Anderson DR. Clinical decisions in glaucoma. St Louis: The CV Mosby Co; 1993. pp. 52–61.

[16] Shroff S, Rao DP, Savoy FM, Shruthi S, Hsu CK, Pradhan ZS, et al. Agreement of a novel artificial intelligence software with optical coherence tomography and manual grading of the optic disc in glaucoma. J Glaucoma. 2023;32:280–286. 10.1097/IJG.0000000000002147 36730188

[17] Landis JR, Koch GG (1977). The measurement of observer agreement for categorical data. Biometrics.

[18] Abràmoff MD, Lavin PT, Birch M, Shah N, Folk JC (2018). Pivotal trial of an autonomous AI-based diagnostic system for detection of diabetic retinopathy in primary care offices. NPJ Digit Med.

[19] Dandona L, Dandona R, Srinivas M, Mandal P, John RK, McCarty CA (2000). Open-angle glaucoma in an urban population in southern India: the Andhra Pradesh eye disease study. Ophthalmology.

[20] Ramakrishnan R, Nirmalan PK, Krishnadas R, Thulasiraj RD, Tielsch JM, Katz J (2003). Glaucoma in a rural population of southern India: the Aravind comprehensive eye survey. Ophthalmology.

[21] Vijaya L, George R, Paul PG, Baskaran M, Arvind H, Raju P (2005). Prevalence of open-angle glaucoma in a rural south Indian population. Invest Ophthalmol Vis Sci.

[22] Tang J, Liang Y, O’Neill C, Kee F, Jiang J, Congdon N (2019). Cost-effectiveness and cost-utility of population-based glaucoma screening in China: a decision-analytic Markov model. Lancet Glob Health.

[23] John D, Parikh R (2017). Cost-effectiveness and cost-utility of community screening for glaucoma in urban India. Public Health.

[24] Shabbir A, Rasheed A, Shehraz H, Saleem A, Zafar B, Sajid M (2021). Detection of glaucoma using retinal fundus images: a comprehensive review. Math Biosci Eng.

[25] Phan S, Satoh SI, Yoda Y, Kashiwagi K, Oshika T (2019). Evaluation of deep convolutional neural networks for glaucoma detection. Jpn J Ophthalmol.

[26] Liu H, Li L, Wormstone IM, Qiao C, Zhang C, Liu P (2019). Development and validation of a deep learning system to detect glaucomatous optic neuropathy using fundus photographs. JAMA Ophthalmol.

[27] Liu S, Graham SL, Schulz A, Kalloniatis M, Zangerl B, Cai W (2018). A deep learning-based algorithm identifies glaucomatous discs using monoscopic fundus photographs. Ophthalmol Glaucoma.

[28] Liu S, Graham SL, Schulz A, Kalloniatis M, Zangerl B, Cai W (2018). Performance of deep learning architectures and transfer learning for detecting glaucomatous optic neuropathy in fundus photographs. Sci Rep.

[29] Shibata N, Tanito M, Mitsuhashi K, Fujino Y, Matsuura M, Murata H (2018). Development of a deep residual learning algorithm to screen for glaucoma from fundus photography. Sci Rep.

[30] Li Z, He Y, Keel S, Meng W, Chang RT, He M (2018). Efficacy of a deep learning system for detecting glaucomatous optic neuropathy based on color fundus photographs. Ophthalmology.

[31] Ting DSW, Cheung CY, Lim G, Tan GSW, Quang ND, Gan A (2017). Development and validation of a deep learning system for diabetic retinopathy and related eye diseases using retinal images from multiethnic populations with diabetes. JAMA.

[32] Chakrabarty L, Joshi GD, Chakravarty A, Raman GV, Krishnadas SR, Sivaswamy J (2016). Automated detection of glaucoma from topographic features of the optic nerve head in color fundus photographs. J Glaucoma.

[33] Issac A, Partha Sarathi M, Dutta MK (2015). An adaptive threshold-based image processing technique for improved glaucoma detection and classification. Comput Methods Prog Biomed.

[34] Ipp E, Liljenquist D, Bode B, Shah VN, Silverstein S, Regillo CD (2021). EyeArt Study Group. Pivotal evaluation of an artificial intelligence system for autonomous detection of referrable and vision-threatening diabetic retinopathy. JAMA Netw Open.

[35] Sosale B, Aravind SR, Murthy H, Narayana S, Sharma U, Gowda SGV (2020). Simple, mobile-based artificial intelligence algorithm in the detection of diabetic retinopathy (SMART) study. BMJ Open Diabetes Res Care.

[36] Sivaraman A, Nagarajan S, Vadivel S, Dutt S, Tiwari P, Narayana S (2021). A novel, smartphone-based, teleophthalmology-enabled, widefield fundus imaging device with an autocapture algorithm. Transl Vis Sci Technol.

[37] Prathiba V, Rajalakshmi R, Arulmalar S, Usha M, Subhashini R, Gilbert CE (2020). Accuracy of the smartphone-based nonmydriatic retinal camera in the detection of sight-threatening diabetic retinopathy. Indian J Ophthalmol.

[38] Sivaprasad S, Netuveli G, Wittenberg R, Khobragade R, Sadanandan R, Gopal B (2021). Nayanamritham Project Collaborators. Complex interventions to implement a diabetic retinopathy care pathway in the public health system in Kerala: the Nayanamritham study protocol. BMJ Open.

[39] Natarajan S, Jain A, Krishnan R, Rogye A, Sivaprasad S (2019). Diagnostic accuracy of community-based diabetic retinopathy screening with an offline artificial intelligence system on a smartphone. JAMA Ophthalmol.

