# Supplementary material for: Evaluation of an offline, artificial intelligence system for referable glaucoma screening using a smartphone-based fundus camera: a prospective study
Source: Eye (Lond). 2023 Dec 13;38(6):1104–11. doi: 10.1038/s41433-023-02826-z (PMC11009383; doi:10.1038/s41433-023-02826-z)
Supplement: Supplementary file 1 — Supplementary methods [file 41433_2023_2826_MOESM1_ESM.docx]

**Supplementary Methods**

**Section 1. Exclusion criteria:**

Participants with acute or sudden vision loss, narrow angles on gonioscopy and who could not be safely dilated, those with co-existing ocular pathologies or significant media opacity precluding adequate view of the disc were excluded. Advanced cataract was defined as the presence of nuclear sclerosis/opalescence grade 3 (NS/NO3) and/or cortical cataract C4 and/or posterior subcapsular cataract P4 based on the Lens Opacities Classification System LOCS III and excluded from the study.^40^ Additionally, subjects with uncontrolled intraocular pressure, active eye infection, who underwent glaucoma filtering surgery less than 3 months before, who had cataract surgery less than one month before, with unstable medical status including blood pressure or glycemic control, photosensitivity etc were excluded. Lastly, subjects were also excluded if any of the imaging modalities showed a failure of quality check due to poor quality images such as being ungradable on image grading or AI quality check failure.

**Section 2. Criteria for final diagnosis following a thorough glaucoma evaluation**

|  | **Normal** | **Suspect** | **Glaucoma** |
| --- | --- | --- | --- |
| **Optic disc exam** | Optic disc with no glaucomatous features | Any one of the following:   1. vCDR >= 0.7 and < 0.9 2. Rim width > 0.05 DD to <= 0.1 DD (between 5-7 o-clock or 11-1 o-clock) 3. Retinal nerve fibre layer defect reaching upto disc 4. Disc Hemorrhage | Any criterion of the following (if subject could not satisfactorily complete a visual field test)   1. vCDR >= 0.9 2. Rim width <= 0.05 DD or localized notches (between 5-7 o-clock or 11-1 o-clock) 3. RNFL defect corresponds to a narrowing of rim/any localized notch   OR  Any one of the following (if subject could satisfactorily complete a visual field test)   1. VCDR > 0.7 2. NRR width <= 0.1 CDR between 5-7 o-clock or 11-1 o-clock 3. Disc haemorrhage 4. RNFLD corresponding to narrow rim or localized notch |
|  | AND | AND | AND |
| **SD-OCT** | RNFL thickness within normal limits for all sectors and global | RNFL <5% (yellow) in either quadrant or clock hour map when segmentation is reliable | RNFL <1% (red) in either quadrant or clock hour map when segmentation is reliable |
|  | AND | AND/OR | AND |
| **Standard Automated Perimetry** | GHT within normal limits and PSD > 5% | Does not meet the criteria for normal or glaucoma | Abnormal but reliable VF with either:   1. GHT outside normal limits, PSD < 5% 2. Localised superior/inferior hemifield loss that correlates with structural damage |

**Section 3. Image quality assessment:** Disc-centered images are graded into:

**Grade 1**- Excellent: Optic disc details (such as the edges and small capillaries) and surrounding RNFL for at least 1DD from the edge of the optic disc are clearly visible to make a diagnosis. 3^rd^ order vessels/capillaries are visualized. Blur/artifacts/glare/over/underexposure in peripheral 1/3^rd^ of image may be disregarded.


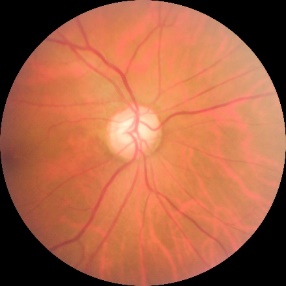

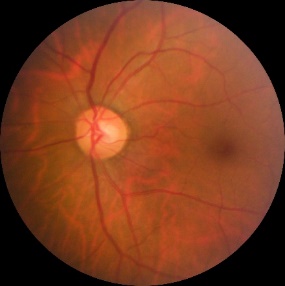

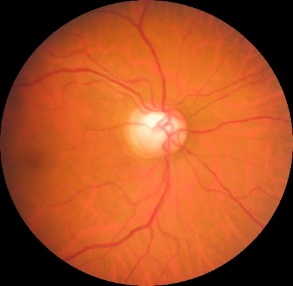


**Excellent images**

**Grade 2**- Acceptable: Optic disc details and surrounding RNFL for at least 1DD from the edge of the optic disc is sufficiently seen to make a diagnosis but not as clearly as compared to an excellent image. 2^nd^order/large blood vessels are visualized. Mild blur/artifacts/glare/ over or under exposure involving 1/3^rd^ to half of image but not affecting optic disc or surrounding RNFL to make a diagnosis is acceptable.


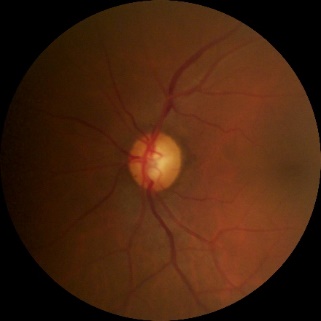

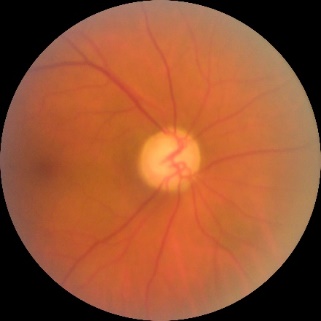

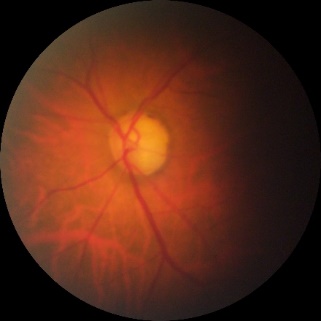


**Acceptable images**

**Grade 3**- Unacceptable: No disc details seen.  Cup margin not made out and peripapillary RNFL region for at least 1DD not seen sufficiently well to make a reliable diagnosis of presence/absence of glaucoma. Retinal vessels not made out.


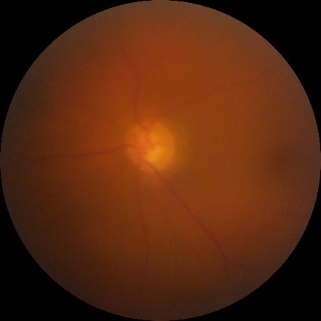

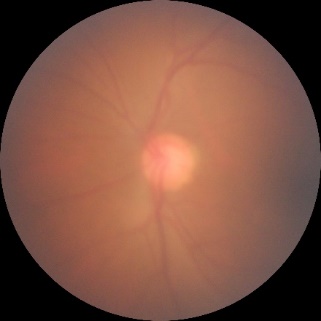

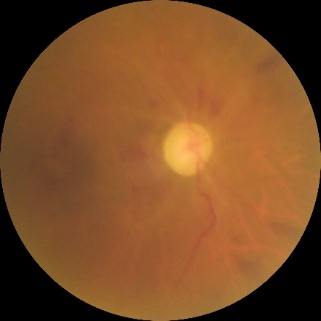


**Ungradable images**

Excellent and acceptable fall under the category of “sufficient'' to make a diagnosis and unacceptable was categorised as “insufficient” to make a glaucoma diagnosis.

**Section 4. Glaucoma diagnosis on image grading based on optic disc evaluation**

- Likely/ Certain glaucoma: Any criterion of the following
  - VCDR >= 0.9
  - Rim width <=0.05 DD or localized notches (between 5-7 o'clock or 11-1 o’ clock)
  - RNFL defect corresponds to a narrowing of the rim
  - RNFL defect corresponds to any localized notch (excavation/complete notch)
- Glaucoma suspect: Any one of these with or without beta peri-papillary atrophy
  - VCDR >= 0.7 - <0.9
  - Rim width > 0.05 DD to <= 0.1 DD (between 5-7 o'clock or 11-1 o'clock)
  - Isolated RNFLD reaching up to disc
  - Disc haemorrhage
- Unlikely glaucoma: If none of the above features is present

Likely glaucoma was categorised as ‘referable glaucoma’ and disc suspect, unlikely glaucoma as ‘no referable glaucoma’.
