# Supplementary figures and images for: Evaluation of an offline, artificial intelligence system for referable glaucoma screening using a smartphone-based fundus camera: a prospective study
Source: Eye (Lond). 2023 Dec 13;38(6):1104–11. doi: 10.1038/s41433-023-02826-z (PMC11009383; doi:10.1038/s41433-023-02826-z)

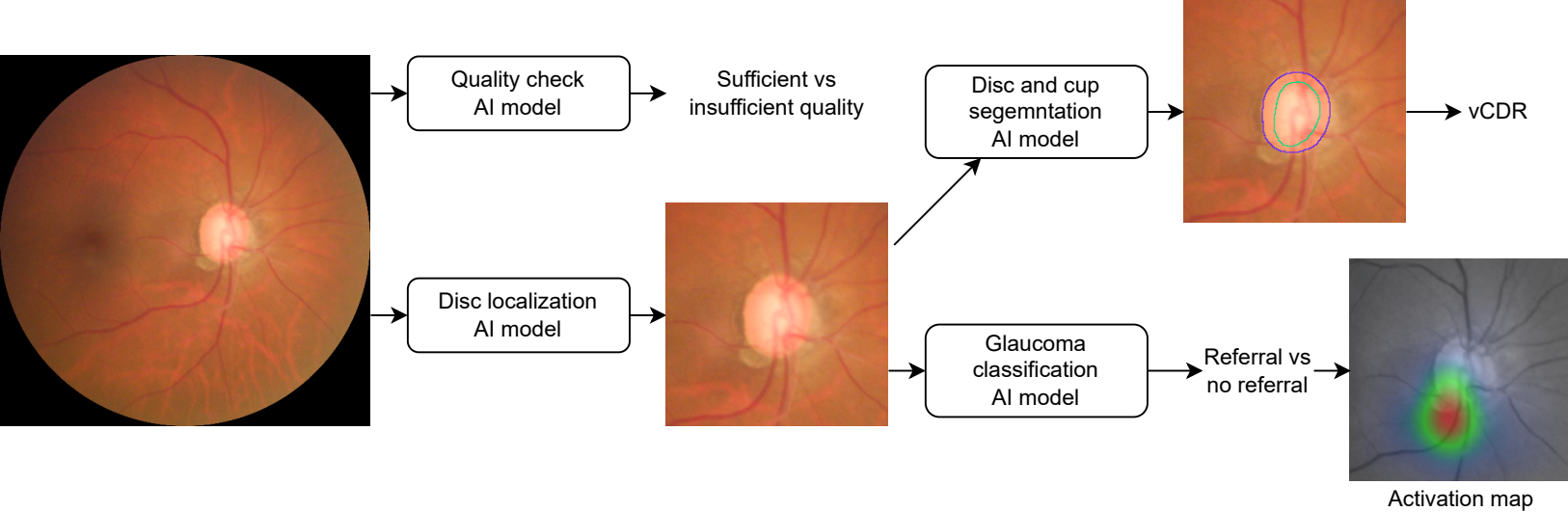

Supplement: Supplementary file 2 — Supplementary Flowchart [file 41433_2023_2826_MOESM2_ESM.pdf]
