# Supplementary material for: Evaluation of an offline, artificial intelligence system for referable glaucoma screening using a smartphone-based fundus camera: a prospective study
Source: Eye (Lond). 2023 Dec 13;38(6):1104–11. doi: 10.1038/s41433-023-02826-z (PMC11009383; doi:10.1038/s41433-023-02826-z)
Supplement: Supplementary file 3 — Supplementary Table 1 [file 41433_2023_2826_MOESM3_ESM.docx]

**Supplementary Table 1: Evaluation of the image quality on FOP by both graders and AI at an eye level**

|  |  | **AI quality check** | | |
| --- | --- | --- | --- | --- |
|  |  | **Sufficient** | **Insufficient** | **TOTAL** |
| **Image grading on FOP** | **Sufficient** | 493 | 29 | 522 |
|  | **Insufficient** | 8 | 15 | 23 |
|  | **No consensus** | 3 | 1 | 4 |
|  | **TOTAL** | 504 | 45 | 549 |
