# Supplementary material for: Evaluation of an offline, artificial intelligence system for referable glaucoma screening using a smartphone-based fundus camera: a prospective study
Source: Eye (Lond). 2023 Dec 13;38(6):1104–11. doi: 10.1038/s41433-023-02826-z (PMC11009383; doi:10.1038/s41433-023-02826-z)
Supplement: Supplementary file 4 — Supplementary table 2 [file 41433_2023_2826_MOESM4_ESM.docx]

**Supplementary Table 2: Summary of Studies Using AI to Detect Glaucoma from Fundus Photographs**

| **Research group and Study design** | **Imaging device** | **Sample (Testing)** | **Race/**  **Ethnicity** | **Artificial Intelligence/ ML Classifier used** | **Performance metrics** |
| --- | --- | --- | --- | --- | --- |
| **Our study (2022)**  **Prospective** | **Remidio NM-FOP** | **504 images from 237 subjects** | **Indian** | **CNN** | **Against standard of care clinical diagnosis-**  Sensitivity 93.7%, Specificity 85.6% |
| Phan et al (2019)  Retrospective ^25^ | Topcon TC50DX and CR2 (Canon) | 2328 images | Japanese | CNN | **Against image grading-**  AUC 0.9 |
| Liu et al (2019)  Retrospective ^26^ | Fundus photographs from Chinese Glaucoma Study Alliance database | 28569 images | Chinese | CNN | **Against image grading-**  AUC 0.996,  Sensitivity 96.2%, Specificity 97.7% |
| Liu et al (2018) Retrospective ^27^ | Different monoscopic fundus cameras | 800 images | White/ Asian | CNN | **Against image grading**-  Accuracy rate - 92.7%  Sensitivity - 89.3%, Specificity - 97.1% |
| Shibata et al (2018)  Retrospective ^29^ | Kowa nonmyd WX camera | 60 eyes of 60 glaucoma patients, 50 eyes of 50 normal subjects | Japanese | CNN | **Against image grading-**  AUC 96.5  (95% CI: 93.5 to 99.6%) |
| Li et el (2018)  Retrospective ^30^ | Online dataset of fundus images LabelMe | 8000 images | Chinese | CNN | **Against image grading-**  AUC of 0.986  Sensitivity 95.6% specificity 92.0% |
| Ting et al (2017)  Retrospective ^31^ | Fundus photographs | 71896 images; 14880 patients | Diverse group | CNN | **Against image grading-**  AROC 0.942;  Sensitivity 96.4%, Specificity 87.2% |
| Chakrabarty et al (2016)  Prospective ^32^ | Topcon TRC50EX fundus camera and  Zeiss Visucam NM/FA fundus camera | 314 eyes  (145 normal, 64 suspect and 105 glaucoma) | Indian | Feature extraction | **Against clinical diagnosis-**  ROC 0.792;  Sensitivity 71.6 % Specificity 71.7% |
| Issac et al (2015)  Retrospective ^33^ | Local database fundus images | 17 images  (7 glaucoma) | Indian | Adaptive threshold-based image processing  (SVM and ANN) | **Against image grading-**  Accuracy 94.11%; Sensitivity of 100% |
